# Supplementary material for: Social contexts and black families’ engagement in early childhood programs
Source: PLoS One. 2025 Jan 31;20(1):e0316680. doi: 10.1371/journal.pone.0316680 (PMC11785340; doi:10.1371/journal.pone.0316680)
Supplement: S1 Appendix — (DOCX) [file pone.0316680.s001.docx]

**S1 Appendix. Study procedure details and supplementary figures and tables**

Section 1: Study 1: Focus Groups

Section 2: Study 2: National Survey

Section 3: Study 3: Field Experiment

Section 4: Studies 2 and 3 Survey Quality Check Procedures

Supplementary Figures and Tables: Figure A, Tables A to K

Supporting Information References

# **Section 1: Study 1. Exploratory Focus Groups with Black Parents**

**Summary**

Study 1 aimed to understand what Black parents and caregivers of young children think about home visiting programs and what informs their decision to participate (or not). Participants were recruited via flyers posted in community centers (e.g., health and recreation centers), email solicitation from community partners (e.g., doula organizations), and a participant pool of local residents who had opted in to receive information about university-led research studies. The study site was chosen because it is one of a growing number of counties to offer universal, community-wide home visits to all families who give birth in county hospitals.

**Focus Group Protocol**

1. Let’s start by introducing ourselves. We’ll go around and share our names, and if you have a child under three, say their name and age. I’ll start!

1. Our first questions today are about how you seek support as parents of infants. There’s so much information out there about what parents “should” and “shouldn’t do”—it can be really overwhelming! We want to hear from you: Who or what are your sources of information about infant care? Can you tell me why you find these sources trustworthy?
2. This question I want everybody to answer. I’m going to ask the question, then you think about it, and keep your answer in your head or write it down. Then we’ll go around and share. What comes to mind when you hear the term “nurse home visits”?
3. What support services for parents of infants were you made aware of during your pregnancy? During your hospital stay? After your hospital stay?

*The moderator then described the home visiting program that operates in the county where the focus group took place, as follows:*

*[Name of the local home visiting program]* is a community-wide nurse home visiting program that aims to bring together families, community agencies, and healthcare providers to provide babies the best environment for growth. The program does this by visiting parents and babies in their own homes. During the home visit, nurses provide a child health check, answer any questions that parents may have, and link families to relevant resources in the community. Depending on the family’s interests and needs, nurses offer help with breastfeeding, childcare resources, recommending financial resources, locating parenting classes, coping with postpartum depression, dealing with social isolation or new family dynamics, and many other issues.

1. If you had known about this, would you have wanted to participate? Why or why not? *[Separate questions were provided for parents who had participated in the program, which applied to only one focus group participant in our study.]*
2. Are there any barriers you would have had to participating? Any additional information you would want to know?
3. Data from the home visiting program show that Black, white, and Latinx parents sign up for home visits in the hospital at similar rates, but Black parents are less likely to confirm their appointment and have the nurse come to their home. Does that surprise you? Why or why not?
4. The key goal of the home visiting program is to connect parents to community resources. In your perspective, what would be some ideal ways to connect parents with community resources?
5. What could the home visiting program do better or do differently to connect with more Black families?
6. Anything else you would like to share before we finish?

# **Section 2: Study 2. Survey of Black Parents’ Perceptions of Home Visiting**

**Summary**

Study 2 draws from an online survey of parents fielded in May 2022. Participants were recruited via CloudResearch (Prime Panels) from across the U.S.; the full sample includes participants from all 50 states plus the District of Columbia. Eligibility requirements were being at least 18 years old, residing in the U.S., having English language reading proficiency, and being a parent or guardian of child(ren) three years old or younger (including expectant parents). The analysis presented in the main text focuses on Black or African American survey participants (n = 163) residing in 35 states. After completing basic demographic screening and survey items about the types of parenting and early childhood support resources they use, participants were asked the open-ended question, “what comes to mind when you hear ‘home visits’?” The wording was randomized, such that one-third of respondents were asked, “what comes to mind when you hear home visits?”; one-third were asked, “what comes to mind when you hear nurse home visits?”; and one-third were asked, “what comes to mind when you hear home visits for parents of young children?” Responses were coded as mentioning surveillance or not. This wording experiment was exploratory and not pre-registered. We therefore focus our analysis on surveillance associations overall (across all conditions) and report differences in surveillance associations across conditions in ***Table C***.

Participants were then shown a brief description of home visiting:

*“Home visits for new and expectant parents aim to provide parents with support to encourage healthy child development, for example by supporting parent and child health; answering parents' questions about caring for their infant and raising young children; supporting parents to ensure their children are ready for school; and connecting families to community resources.”*

Following this description, participants were asked if they had ever participated in a home visiting program, trust-related barriers to engagement (based on Dumas et al. [1]), and help-seeking stigma [2, 3]. Participants also completed additional demographic questions related to socioeconomic status and country of origin, among others.

**Measures**

**Independent Variable: Surveillance Fears**

Open-ended responses were coded as mentioning surveillance fears if they indicated discomfort or fear (e.g., “intrusive,” “in trouble”), judgment of parenting skills (e.g., “someone coming into your home to determine if you are raising your child right”), or child removal (e.g., “taking children away”), or if they named CPS or other state-specific child welfare agencies. Coding was conducted independently by two research assistants with high inter-rater reliability (kappa = 0.70), and all inconsistencies were reviewed until consensus was achieved. Our analysis focuses on the sub-sample of Black participants, but as shown in ***Figure A***, associating the term “home visits” with surveillance was common across racial and ethnic groups.

Examples of responses coded as fear of surveillance include:

- - *“Social services coming to intrude on a person’s privacy.”*
  - *“Social workers coming to check to see if you are fit to be a parent. Not a good thing, unless it’s actually saving the kid. I would not want a home visit [to have to] prove to someone I take care of my child.”*
  - *“Worrying I did something wrong to initiate a visit.”*
  - *“Having an inspection done to judge the fitness of a parent and the pain of losing someone due to these procedures, and having those procedures fail me, the phrase makes me ill.”*

Examples of responses coded as not representing surveillance fears include:

- - *“I like the idea, of a teacher or somebody checking on my child and me and seeing how I’m doing, I am a first-time mom, so I still need advice and somebody to see how am I doing or just to give me extra advice.”*
  - *“In my mind, home visits include someone coming to check in, hear from and learn about how the past week or so has gone. It reflects on opportunities for parental growth and child health to prosper.”*
  - *“A beneficial and valuable service paid for by many health insurance companies.”*
  - *“Resources and services that come to my home to help with my child.”*

**Dependent Variables**

***Prior participation in home visiting programs.*** Prior participation was coded as 1 if the respondent reported having participated in any home visiting program before, and 0 if not.

***Trust-related obstacles to engagement.*** Trust-related obstacles to engagement were measured using items from the Obstacles to Engagement scale [1]. This scale asks, “What might prevent you from engaging in home visiting?” The trust-related subscale includes five items: (1) lack of trust, (2) feeling frightened or nervous, (3) not wanting to talk about parenting with a stranger, (4) having a stranger in your home, and (5) fear of being judged. Response options range from 1 (definitely not) to 4 (definitely yes), and the scale score was constructed by taking the mean across all five items (*M* = 2.48, *SD* = 0.87, *Min* = 1, *Max* = 4, Cronbach’s alpha = 0.87). Regression models used the within-sample standardized score.

**Covariates**

***Demographics.*** Self-reported educational attainment was coded as 1 for bachelor’s degree or more, and 0 if less than a bachelor’s degree. We operationalized low-income status via self-reported annual household income, wherein incomes less than $40,000 were coded as 1 and incomes above $40,000 were coded as 0. Regarding housing stability, the measure was coded as 1 for those who reported having a steady place to live and 0 for those who did not have stable housing or were worried about losing their current housing.

***Help-seeking stigma.*** Help-seeking stigma captures participants’ fears of judgment for seeking additional help with parenting-related tasks. Help-seeking stigma was measured using the short (3-item version) of Vogel et al.'s 2006 Self-Stigma of Seeking Help (SSOH) scale [3]). In line with prior work using the SSOH to examine stigma for seeking help from parenting programs [4], we adapted the SSOH by changing “therapist” to “home visiting.” Participants were asked, “thinking about home visit programs, how much do you agree or disagree with the following statements?” Statements asked if participants would feel (1) inadequate, (2) inferior, or (3) less satisfied with themselves if they sought support from a home visiting program. Response options ranged from 1 (“strongly disagree”) to 5 (“strongly agree”), and the three items were summed and then standardized for use in our regression models (*M* = 2.61, *SD* = 1.12, *Min* = 1, *Max* = 5, Cronbach’s alpha = 0.90).

**Statistical Analysis**

Study 2 estimates the relation between surveillance fears and home visit prior participation and trust-related obstacles to engagement using the below exploratory (not pre-registered) regression models:

$$Eq. 1. y_{i}= \beta_{0}+ \beta_{1}{Surveillance Association}_{i}+\varepsilon_{i}$$

$$Eq. 2. y_{i}= \beta_{0}+ \beta_{1}S{urveillance Association}_{i} {+ X}_{i}+ \varepsilon_{i}$$

$\beta_{1}$ is the coefficient of interest in both equations, describing the estimated effect of associating home visiting programs with surveillance on dependent variable *y* (self-reported prior participation in a home visiting program and trust-related obstacles to engagement in home visiting) for individual *i*. Equation 2 adjusts for the demographic covariates described above and the home visit wording condition (i.e., “nurse home visits” or “home visits for parents of young children” versus “home visits”), denoted by *X_i_*. Separate regression models were estimated for each of the dependent variables.

# **Section 3: Study 3. Pre-registered Field Experiment**

Link to pre-registration: <https://osf.io/gvw2s>

**Summary**

Study 3 used a 2×2 between-person experimental design, with the following four conditions:

- Condition 1: Surveillance news story + “new baby wellness” label
- Condition 2: Surveillance news story + “home visit” label
- Condition 3: Child-centric control article + “new baby wellness” label
- Condition 4: Child-centric control article + “home visit” label

**Experimental Protocol**

Participants were randomly assigned to read one of the below (fictional) news stories and then completed a timed reflection (shown below). After the reflection, participants read about a home visiting program, with random assignment to the “home visit” or “new baby wellness” label, and then completed survey items assessing interest in learning more and engaging in home visiting (described below), trust-related obstacles to engagement, prior participation in home visiting, help-seeking stigma, experiences of discrimination in medical settings, and demographic information. The randomization process achieved balance on observable characteristics, as shown in ***Table E***.

***Article 1 (Experimental Group): News story about unsubstantiated reports of child abuse designed to cue surveillance fears:***

When child protective services investigated Destiny Johnson’s family in 2022, she could measure the personal toll in pounds lost: 20. She tried to fight the fear that her caseworker “could try and snatch my kids,” a vision she says she still can’t escape in her nightmares. The agency eventually found no evidence her children were being neglected or abused —but the process of having a caseworker inspect their apartment and ask prying questions deeply disturbed her children, who are now 3, 5, and 10.

The family is among the thousands of households across the US subjected to unfounded investigations into abuse or neglect initiated by calls from their children’s school. Many states require educators to call a hotline if they believe a young person may be experiencing abuse or neglect. But, in practice, that decision is always a judgment call. Of the total investigations from 2019, only 16% found evidence of abuse or neglect.

***Article 2 (Control Group): Child-centric control condition story about excessive communication from schools:***

When she received yet another mass email from her child’s preschool, Destiny Johnson was not surprised. In the past week she had already received 9 emails, 5 text messages, and 2 newsletters from her child’s school. She tried to muster the motivation to read yet another message, knowing that amidst the many irrelevant reminders, there may be useful information.

Ms. Johnson is among the thousands of parents across the US subjected to a huge amount of emails, texts, and phone calls from their children’s school. Like many schools, her child’s preschool had experimented with several different apps meant to streamline communication with parents, but none had stuck. Schools deliver information in multiple ways to keep families informed...but do they need to do this much communication? For parents and guardians, managing school chatter can feel like a full-time job.

***Reflection***

How would you feel if you were the parent in this news article? Write 3-4 sentences describing your feelings.

*Click the blue button to move on when you have answered the prompt. The blue button will appear after 60 seconds, but you may spend as much time as you would like to answer the prompt.*

***New Baby Wellness Label***

New Baby Wellness Visits offer support for all families. New babies don’t come with instructions. Anyone who is pregnant or has a young child at home could use some support. New Baby Wellness programs will come to you, listen to you, and build on your families’ existing knowledge, skills, and values to help your child be healthy, safe, and ready to learn.

***Home Visiting Label***

Home visiting offers support for all families. New babies don’t come with instructions. Anyone who is pregnant or has a young child at home could use some support. Home visiting programs will come to you, listen to you, and build on your families’ existing knowledge, skills, and values to help your child be healthy, safe, and ready to learn.

**Measures**

***Dependent Variables***

***Interest in learning more and participating in parenting programs.*** Interest was measured through two items. First, participants were asked, “How interested would you be in learning about [“Home Visiting” or “New Baby Wellness”] programs?” The specific wording depended on the label group to which they were randomly assigned. To follow, participants were asked, “How interested would you be in participating in [“Home Visiting” or “New Baby Wellness”] programs?” In both cases, responses ranged from 0 (“not at all interested”) to 100 (“very interested”).

***Behavioral measure (click-throughs).*** Participants were asked, “would you like to receive information about [“Home Visiting” or “New Baby Wellness”] programs? If yes, please click here [*link to a website listing resources for finding home visiting programs and other resources:* [*https://nhvrc.org/what-is-home-visiting/*](https://nhvrc.org/what-is-home-visiting/)]”

***Covariates***

Covariates include age (indicator variables for 18–25, 26–45, and 36–45, versus older than 45 as the reference category), household size, educational attainment (bachelor’s degree or higher versus less), number of children, household income (indicator variables for $80k+ and $40k–$79k, with less than $40k as the reference category), immigration (born in the U.S. versus not), and region (indicator variables for the West, Midwest, and Northeast, with South as the reference category).

**Moderators**

***Help-seeking stigma.*** Stigma associated with seeking help is measured via the short (3-item version) of Vogel et al.'s Self-Stigma for Seeking Help scale [3] (*M* = 2.04, *SD* = 0.82, *Min* = 1, *Max* = 5, Cronbach’s alpha = 0.79) as described in Study 2. In regression analyses of moderation, the index was dichotomized based on the average index score, such that participants with scores at or above the average index score were coded as 1 (high stigma) and those with below average scores were coded as 0 (low stigma).

***Prior home visit experience.*** This measure was the same as in Study 2.

***Experiences of discrimination in medical settings.*** We used the Everyday Discrimination Scale [5, 6], adapted to reflect experiences of discrimination in medical settings. Participants were asked, “when you interact with medical professionals (doctors, nurses, midwives, other medical staff), how often do any of the following things happen to you?” The list of experiences included five items (e.g., “you received poorer service than other people at the clinic or hospital”). Response options ranged from 0 (“never”) to 4 (“almost always”). The five items in the scale have high internal reliability with a Cronbach’s alpha of 0.87 (*M* = 1.08, *SD* = 0.87, *Min* = 0, *Max* = 5). The five items were averaged and then dichotomized for use in regression analyses of moderation, such that high discrimination (average or above average index score) = 1 and low discrimination (below average index score) = 0.

***Gender.*** Participants self-reported their gender (75% female, 14% male, <1% non-binary). Moderation analyses compared female versus male and non-binary participants.

***Educational attainment.*** Self-reported educational attainment was dichotomized for moderation analyses to high school or less (33%) versus those with greater than a high school education (66%).

***Financial strain.*** Participants were asked, “during a typical week, how often do you worry about being able to meet your monthly living expenses?” Response options included “never,” “rarely,” “sometimes,” or “frequently.” Moderation was tested by collapsing this measure into a dichotomous indicator: 0 = never, rarely, or sometimes and 1 = frequently.

**Mediator: Trust-Related Obstacles to Engagement**

This measure was the same as in Study 2 (*M* = 2.37, *SD* = 0.75, *Min* = 1, *Max* = 4, Cronbach’s alpha = 0.77).

**Pre-registered Analysis Plan**

To estimate the main effects of each experimental condition, we estimated the following equations:

Equation 1, estimated on the sub-sample of participants assigned to Condition 2 and Condition 4, estimates the effect of priming fears of surveillance via the news story about one family’s contact with Child Protective Services due to an unsubstantiated report of child abuse, as follows:

$$Eq. 1. y_{i}= \beta_{0}+ \beta_{1}{Fear of Surveillance}_{i} {+ X}_{i}+ \varepsilon_{i}$$

wherein $\beta_{1}$ estimates the effect of reading about unsubstantiated reports of child abuse (versus the control article) on $y$ (interest in learning more, participating in, and clicking to learn more about home visiting). The subscript *i* indexes participants, and $X$ is a vector of individual-level covariates. Three separate regression analyses were used for each dependent variable.

Equation 2, estimated on the subsample of participants assigned to Condition 3 and Condition 4, estimates the effect of the “new baby wellness” label versus the “home visiting” label:

$$Eq. 2. y_{i}= \beta_{0}+ \beta_{1}{NewBabyWellness}_{i} {+ X}_{i}+ \varepsilon_{i}$$

Equation 3, estimated on the subsample of participants assigned to Condition 1 and Condition 4, estimates the combined effect of both experimental conditions versus the control conditions:

$${Eq.3. y}_{i}= \beta_{0}+ \beta_{1}{Fear of Surveillance+NewBabyWellness}_{i} {+ X}_{i}+ \varepsilon_{i}$$

Finally, Equations 4 and 5 pool the data to test the incremental effects of each experimental condition, as follows:

$$Eq. 4. y_{i}= \beta_{0}+ \beta_{1}{{Fear of Surveillance}_{i}}_{i}+ \beta_{2}{NewBabyWellness}_{i} {+ X}_{i}+ \varepsilon_{i}$$

$$Eq. 5. y_{i}= \beta_{0}+ \beta_{1}{{Fear of Surveillance}_{i}}_{i}+ \beta_{2}{NewBabyWellness}_{i}+ {\beta_{3}{{Fear of Surveillance}_{i}}_{i}*{NewBabyWellness}_{i}+ X}_{i}+ \varepsilon_{i}$$

Equation 4 tests the equality of coefficients $\beta_{1}$ and $\beta_{2}$, i.e., whether there is a statistically significant difference in the effect of priming fears of surveillance versus the effect of the “new baby wellness” label on interest in home visiting.

Finally, Equation 5 tests the interaction between the two experiments. The main coefficient of interest is $\beta_{3}$, which estimates whether the “new baby wellness” versus “home visiting” label buffers the effect of priming fear of surveillance on interest in learning more and participating in home visiting programs.

Results from Equations 1–4 are shown in ***Table G***, and Equation 5 results are presented in ***Table F*** and in the main text.

The above analyses (Equations 1–5) were pre-registered. In addition, we report pre-registered analyses to examine moderation by (1) help-seeking stigma (using the same scale described in Section 2 [2–4]), (2) prior experience with home visits, (3) discrimination in medical settings using an adapted Everyday Experiences of Discrimination scale [5, 6], (4) educational attainment, (5) gender, and (6) financial strain. Moderation was examined via interaction terms of the treatment indicators with the hypothesized moderator of interest (see ***Table H*** and ***Table I*** for marginal effects for statistically significant moderators).

We also report pre-registered analyses examining the role of trust-related obstacles to engagement (adapted from Dumas et al. [1]) as a mediator of the effects of fears of surveillance and “new baby wellness” label on interest in learning more and participating in home visiting programs. Mediation was analyzed in two ways. First, we estimated Equation 5 above, but with trust-related obstacles to engagement as the dependent variable. This model provides an estimate of the effect of reading about unsubstantiated reports of child abuse (surveillance prime) and the “new baby wellness” label on our hypothesized mediator: trust-related obstacles to engagement. As shown in ***Table J***, the “new baby wellness” label reduced trust-related obstacles to engagement, but there was no evidence of an effect of the surveillance prime on trust-related obstacles to engagement. Next, we estimated Equation 5 with trust-related obstacles to engagement included as an independent variable. These regressions (M3–M7 in ***Table J***) provide suggestive evidence for mediation, given that the inclusion of trust-related obstacles to engagement reduced the magnitude (and statistical significance) of the effects of the surveillance prime and “new baby wellness” label on interest in learning more and interest in participating in home visiting.

Results from the pre-registered structural equation model of mediation provide more rigorous evidence for mediation. We estimated two models. The first included the main effect of both treatments and the interaction term (surveillance prime × “new baby wellness” label), the hypothesized mediator, and all three dependent variables. This model fit well ($\chi_{2}$(18) = 1,608.00, *p* < 0.001; AIC = 2,6006.90; BIC = 2,6136.86; CFI = 1.00; TLI = 1.00; RMSEA = 0.000; SRMR = 0.000). In the second model, we dropped the statistically insignificant pathways (i.e., all paths leading from the interaction between the surveillance prime × “new baby wellness” label and all paths leading to the third dependent variable, click-throughs) for model parsimony. This model achieved better fit, per the AIC and BIC fit criterion measures ($\chi_{2}$(9) = 1,559.20, *p* < 0.001; AIC = 2,5161.53; BIC = 2,5236.50; CFI = 1.00; TLI = 1.00; RMSEA = 0.000; SRMR = 0.000) and was retained for analysis (***Figure 1*** in the main text and ***Table K***).

**Deviation from Pre-registration**

The pre-registered analysis plan included age of youngest child as a covariate in Equations 1–5, but this variable was not included in the survey and thus we are unable to include it in the regression models.

# **Section 4: Studies 2 and 3 Survey Quality Check Procedures**

Online survey participants were excluded from analysis if their responses exhibited at least two of the following:

1. Total survey duration less than 2 seconds per question

2. Failing at least 2 of the 4 embedded attention checks (e.g., “select ‘never’ to show you are actively participating”)

3. Evidence of flatlining at least 4 scales

Participants who wrote gibberish, nonsense, or illogical answers to open-ended questions were also excluded (**for Study 3, these exclusion criteria were pre-registered**).

# **Supplementary Figures and Tables**

***Figure A. Study 2 association of the term “home visits” with surveillance, by race and ethnicity***

***Table A. Study 1 participant demographics***

|  | *N* | % |
| --- | --- | --- |
| Gender |  |  |
| Female | 20 | 77% |
| Male | 6 | 23% |
| Non-binary | 1 | 4% |
| Parent/caregiver type |  |  |
| Currently expecting first child | 6 | 23% |
| Have a child <3 years old | 17 | 65% |
| Have a child <3 years old and currently expecting | 4 | 15% |
| Age |  |  |
| 18–24 | 13 | 50% |
| 25–34 | 10 | 38% |
| 35–44 | 4 | 15% |
| Hispanic ethnicity |  |  |
| Yes | 11 | 42% |
| No | 16 | 62% |
| Educational attainment |  |  |
| High school diploma or GED | 2 | 8% |
| Some college, no degree | 3 | 12% |
| Associate degree | 6 | 23% |
| Bachelor's degree | 15 | 58% |
| Master's or professional degree | 1 | 4% |
| Annual household income |  |  |
| Less than $10k | 1 | 4% |
| $10k–$19k | 1 | 4% |
| $20k–$29k | 0 | 0% |
| $30k–$39k | 8 | 31% |
| $40k–$49k | 5 | 19% |
| $50k–$59k | 7 | 27% |
| $60k–$69k | 4 | 15% |
| $70k–$79k | 1 | 4% |
| $80k+ | 0 | 0% |
| *Total* | 26 | 100% |

***Table B. Study 2 participant demographics***

|  | Mean |
| --- | --- |
| **Demographics** |  |
| Black or African American | 100% |
| Multiracial (Black or African American and some other race): | 18% |
| 1. American Indian or Alaskan Native | 4% |
| 2. Asian American or Pacific Islander | 1% |
| 3. White | 7% |
| 4. Other race | 1% |
| Hispanic or Latino | 11% |
| Pregnant or expecting first child | 3% |
| Total number of children | 1.96  (1.15) |
| Gender: female | 76% |
| Gender: male | 24% |
| Gender: non-binary | 1% |
| Age in years | 33.31  (8.38) |
| **Socioeconomic Characteristics** |  |
| Household size (number of people) | 3.80  (1.35) |
| Currently working | 63% |
| *Housing stability* |  |
| I have a steady place to live | 79% |
| I have a steady place to live but I am worried about losing it | 18% |
| I do not have a steady place to live | 3% |
| *Educational attainment* |  |
| Less than high school | 1% |
| Some high school | 3% |
| High school diploma | 29% |
| GED or equivalent | 3% |
| Some college | 29% |
| Associate degree | 7% |
| Bachelor’s degree | 20% |
| Master’s or professional degree | 6% |
| Doctorate | 1% |
| *Annual household income* |  |
| $10k or less | 10% |
| $11k–$20k | 15% |
| $21k–$30k | 15% |
| $31k–$40k | 10% |
| $41k–$50k | 15% |
| $51k–$60k | 12% |
| $61k–$70k | 7% |
| $71k–$80k | 3% |
| $80k or more | 12% |
| *N* | 163 |

*Note*: The table shows percentages for bivariate variables and means with standard deviations in parentheses for continuous variables.

***Table C. Study 2 effect of home visit wording on surveillance fear***

|  | Surveillance Fear |
| --- | --- |
| Wording 1: “nurse home visits” | -0.07 |
|  | (0.08) |
| Wording 2: “home visits for parents of young children” | 0.22** |
|  | (0.08) |
| Constant | 0.18** |
|  | (0.06) |
| *N* | 163 |
| Adjusted R-squared | 0.086 |

*Note*: OLS regression analysis. The table shows coefficients with standard errors in parentheses.

The reference category wording is “home visits.”

* *p* < .05, ** *p* < .01, *** *p* < .001

***Table D. Study 2 relation between surveillance fear and home visiting participation and engagement***

|  | Prior Participation in Home Visiting | | Trust-Related Obstacles to Engagement | |
| --- | --- | --- | --- | --- |
|  | (1) | (2) | (3) | (4) |
| Surveillance fear | -0.183* | -0.188* | 0.399* | 0.361* |
|  | (0.0837) | (0.0872) | (0.183) | (0.180) |
|  |  |  |  |  |
| Educational attainment |  | 0.135 |  | -0.342 |
|  |  | (0.0898) |  | (0.186) |
|  |  |  |  |  |
| Help-seeking stigma |  | 0.0299 |  | 0.303*** |
|  |  | (0.0357) |  | (0.0740) |
|  |  |  |  |  |
| Low income |  | -0.212* |  | 0.0483 |
|  |  | (0.0815) |  | (0.169) |
|  |  |  |  |  |
| Housing stability |  | -0.111 |  | -0.619** |
|  |  | (0.0934) |  | (0.195) |
|  |  |  |  |  |
| Label: "nurse home visits" |  | 0.0379 |  | -0.320 |
|  |  | (0.0898) |  | (0.187) |
|  |  |  |  |  |
| Label: "home visits for parents of young children" |  | 0.0546 |  | -0.275 |
|  |  | (0.0887) |  | (0.185) |
|  |  |  |  |  |
| Constant | 0.358*** | 0.482*** | -0.0558 | 0.725** |
|  | (0.0415) | (0.124) | (0.0907) | (0.261) |
|  |  |  |  |  |
| *N* | 163 | 159 | 162 | 158 |
| Adjusted R-squared | 0.023 | 0.097 | 0.023 | 0.187 |

*Note*: OLS regression analysis. The table shows coefficients with standard errors in parentheses. Prior participation in home visiting is binary (interpreted as a linear probability model), with no=0, yes =1. Trust-related obstacles to engagement is standardized.

* *p* < .05, ** *p* < .01, *** *p* < .001

***Table E. Study 3 participant demographics and balance across experimental conditions***

|  | Full Sample | Condition 1: Surveillance News Story × “New Baby Wellness” Label | Condition 2: Surveillance News Story × “New Baby Wellness” Label | Condition 3: Surveillance News Story × “New Baby Wellness” Label | Condition 4: Surveillance News Story × “New Baby Wellness” Label |
| --- | --- | --- | --- | --- | --- |
|  |  |  |  |  |  |
| Monoracial Black | 0.92 | 0.95 | 0.90 | 0.92 | 0.92 |
| Multiracial Black | 0.08 | 0.05 | 0.10 | 0.08 | 0.08 |
| Female | 0.75 | 0.75 | 0.77 | 0.75 | 0.74 |
| Male | 0.24 | 0.25 | 0.23 | 0.24 | 0.26 |
| Non-binary | 0.00 | 0.01 | 0.00 | 0.01 | 0.00 |
| Hispanic or Latino | 0.09 | 0.05 | 0.11 | 0.07 | 0.11 |
| Born in the U.S. | 0.96 | 0.94 | 0.96 | 0.96 | 0.97 |
|  |  |  |  |  |  |
| Age | 31.87 | 31.62 | 31.66 | 32.57 | 31.66 |
| Number of children | 2.35 | 2.26 | 2.44 | 2.37 | 2.35 |
| Expecting | 0.27 | 0.31 | 0.27 | 0.27 | 0.23 |
| Pregnant | 0.16 | 0.17 | 0.17 | 0.17 | 0.13 |
| Household size | 3.94 | 3.93 | 4.10 | 3.89 | 3.86 |
|  |  |  |  |  |  |
| Less than high school | 0.00 | 0.00 | 0.01 | 0.00 | 0.00 |
| Some high school | 0.03 | 0.02 | 0.03 | 0.03 | 0.04 |
| High school diploma | 0.26 | 0.25 | 0.27 | 0.25 | 0.28 |
| GED or equivalent | 0.04 | 0.04 | 0.03 | 0.04 | 0.04 |
| Some college no degree | 0.28 | 0.34 | 0.22 | 0.30 | 0.25 |
| Associate degree | 0.15 | 0.12 | 0.21 | 0.12 | 0.14 |
| Four-year degree (BA or BS) | 0.17 | 0.17 | 0.17 | 0.17 | 0.18 |
| Master’s or professional degree | 0.05 | 0.05 | 0.03 | 0.06 | 0.06 |
| Doctorate | 0.02 | 0.01 | 0.03 | 0.02 | 0.02 |
|  |  |  |  |  |  |
| Low income (less than $40k) | 0.42 | 0.45 | 0.39 | 0.43 | 0.40 |
| Medium income ($40k–$80k) | 0.31 | 0.30 | 0.31 | 0.31 | 0.34 |
| High income ($80k or more) | 0.14 | 0.10 | 0.15 | 0.17 | 0.15 |
| Worry about meeting bills never or rarely | 0.23 | 0.24 | 0.23 | 0.21 | 0.24 |
| Worry about meeting bills sometimes | 0.38 | 0.35 | 0.41 | 0.41 | 0.37 |
| Worry about meeting bills frequently | 0.39 | 0.41 | 0.36 | 0.38 | 0.40 |
| Prior participation in home visiting | 0.11 | 0.11 | 0.09 | 0.12 | 0.12 |
| Region: West | 0.11 | 0.10 | 0.10 | 0.13 | 0.12 |
| Region: Midwest | 0.16 | 0.16 | 0.18 | 0.16 | 0.14 |
| Region: South | 0.58 | 0.59 | 0.60 | 0.53 | 0.60 |
| Region: Northeast | 0.14 | 0.14 | 0.12 | 0.18 | 0.14 |
| *N* | *1,097* | *295* | *263* | *252* | *287* |
| *F-test of joint orthogonality* |  |  |  |  |  |
| Condition 1 vs. 2 | F(13, 451) = 1.32; *P* > F = 0.20 | |  |  |  |
| Condition 1 vs. 3 | F(13, 455) = 0.96; *P* > F = 0.49 | |  |  |  |
| Condition 1 vs. 4 | F(13, 481) = 1.49; *P* > F = 0.12 | |  |  |  |
| Condition 2 vs. 3 | F(13, 426) = 1.26; *P* > F = 0.24 | |  |  |  |
| Condition 2 vs. 4 | F(13, 452) = 0.79; *P* > F = 0.67 | |  |  |  |
| Condition 3 vs. 4 | F(13, 456) = 0.97; *P* > F = 0.48 | |  |  |  |

*Note*: The table shows sample mean demographic characteristics for the full sample and each treatment condition. F-tests of joint orthogonality test for balance in observable characteristics across treatment groups by estimating linear regression models of treatment on demographic characteristics (multiracial identity; Hispanic; U.S. born; female gender; household size; number of children; expecting or pregnant; bachelor’s degree or higher educational attainment; household income $40k or less; West, Midwest, or Northeast location), separately for each of the treatment contrasts, as shown above.

***Table F. Study 3 experimental effects of a news story designed to prime surveillance fears (versus a neutral child-centric news story) and “new baby wellness” (versus “home visiting”) label on Black parents’ interest in home visiting programs***

|  | Interest in Learning More | | Interest in Participating | | Clicked on Link to Learn More | |
| --- | --- | --- | --- | --- | --- | --- |
|  | (1) | (2) | (3) | (4) | (5) | (6) |
| Surveillance news story | -4.342* | -4.974* | -3.610 | -5.000* | -0.036 | -0.028 |
|  | (2.484) | (2.658) | (2.467) | (2.657) | (0.030) | (0.034) |
|  |  |  |  |  |  |  |
| “New baby wellness” label | 8.313*** | 8.975*** | 10.626*** | 11.945*** | 0.012 | 0.007 |
|  | (2.512) | (2.637) | (2.495) | (2.636) | (0.030) | (0.033) |
|  |  |  |  |  |  |  |
| Surveillance news story × “new baby wellness” label | 5.551 | 5.746 | 0.802 | 1.182 | 0.044 | 0.046 |
|  | (3.521) | (3.754) | (3.498) | (3.752) | (0.042) | (0.048) |
| Control group mean | 62.00 | | 68.92 | | 0.162 | |
| Covariates | No | Yes | No | Yes | No | Yes |
| *N* | *1,097* | *942* | *1,097* | *942* | *1,097* | *942* |
| Adjusted R-squared | 0.035 | 0.070 | 0.034 | 0.075 | 0.001 | 0.028 |

*Note*: OLS regression models. The table shows coefficients with standard errors in parentheses. Models represent Equation 5 in the pre-registered analysis plan (see **Section 3** and OSF pre-registration: https://osf.io/tv2s6/). Consistent with our pre-registered directional hypotheses, *p*-values for one-sided hypotheses are reported.

* *p* < .05, ** *p* <.01, *** *p* < .001

***Table G. Study 3 pre-registered analyses of experimental effects***

| ***Eq. 1: Experimental Contrast 1*** | |  |  |  | ***Eq. 2: Experimental Contrast 2*** | |  |  |
| --- | --- | --- | --- | --- | --- | --- | --- | --- |
|  | Interest in Learning More | Interest in Participating | Clicked on Link to Learn More |  |  | Interest in Learning More | Interest in Participating | Clicked on Link to Learn More |
| Surveillance news story | -5.045+ | -5.008+ | -0.0269 |  | “New baby wellness” label | 8.962*** | 12.12*** | 0.00833 |
|  | (2.788) | (2.818) | (0.0324) |  |  | (2.625) | (2.620) | (0.0335) |
| Constant | 77.52*** | 82.24*** | 0.238* |  | Constant | 62.86*** | 62.20*** | 0.236+ |
|  | (11.43) | (11.56) | (0.133) |  |  | (9.721) | (9.704) | (0.124) |
| *N* | *470* | *470* | *470* |  | *N* | *476* | *476* | *476* |
| Adjusted R-squared | 0.080 | 0.075 | 0.028 |  | Adjusted R-squared | 0.038 | 0.069 | 0.037 |
|  |  |  |  |  |  |  |  |  |
| ***Eq. 3: Experimental Contrast 3*** | | |  |  | ***Eq. 4: Differential Effects of Contrasts 1 and 2*** | | | |
|  | Interest in Learning More | Interest in Participating | Clicked on Link to Learn More |  |  | Interest in Learning More | Interest in Participating | Clicked on Link to Learn More |
| Surveillance news story & “new baby wellness” label | 9.335*** | 7.479** | 0.0203 |  |  |  |  |  |
|  | (2.590) | (2.667) | (0.0337) |  | Surveillance news story | -2.104 | -4.410* | -0.00519 |
| Constant | 61.28*** | 71.83*** | 0.353** |  |  | (1.885) | (1.882) | (0.0239) |
|  | (9.724) | (10.01) | (0.127) |  |  |  |  |  |
| *N* | *500* | *500* | *500* |  | “New baby wellness” label | 11.80*** | 12.53*** | 0.0300 |
| Adjusted R-squared | 0.048 | 0.038 | 0.048 |  |  | (1.885) | (1.882) | (0.0239) |
|  |  |  |  |  | Constant | 66.10*** | 67.02*** | 0.317*** |
|  |  |  |  |  |  | (7.191) | (7.179) | (0.0912) |
|  |  |  |  |  | *N* | *942* | *942* | *942* |
|  |  |  |  |  | Adjusted R-squared | 0.069 | 0.076 | 0.028 |

*Note*: OLS regression models. The table shows coefficients with standard errors in parentheses. All models include pre-registered covariates. See details of sub-samples for each equation in **Section 3** and the OSF pre-registration. In Eq. 4, the effect of the surveillance news story is statistically different from the effect of the “new baby wellness” label on interest in learning more (F(1, 925) = 25.64, *p* < .001) and interest in participating (F(1, 925) = 38.17, *p* < .001), but not on click-through (F(1,925) = 1.02, *p* = .313). Additionally, the magnitudes of the coefficients for both experimental conditions were statistically different for interest in learning more (F (1, 925) = 14.08, *p* < .001) and interest in participating (F(1, 925) = 9.90, *p* = .002) (assessed by recoding the label variable such that 1 = “home visiting” and 0 = “new baby wellness,” to ensure both coefficients would be in the same direction).

+ *p* < .10, * *p* < .05, ** *p* <.01, *** *p* < .001

***Table H. Study 3 pre-registered moderation of experimental effects***

**Panel A: Moderation by gender, education, and financial strain**

|  | Gender (Female) | | |  | Educational Attainment  (High School or Less) | | |  | Financial Strain | | |
| --- | --- | --- | --- | --- | --- | --- | --- | --- | --- | --- | --- |
|  | Interest in Learning More | Interest in Participating | Clicked on Link to Learn More |  | Interest in Learning More | Interest in Participating | Clicked on Link to Learn More |  | Interest in Learning More | Interest in Participating | Clicked on Link to Learn More |
| Moderator | -4.557 | -4.059 | 0.035 |  | -1.670 | -4.618 | -0.046 |  | -1.457 | -2.195 | -0.021 |
|  | (3.675) | (3.680) | (0.049) |  | (3.382) | (3.372) | (0.042) |  | (3.303) | (3.299) | (0.042) |
|  |  |  |  |  |  |  |  |  |  |  |  |
| Surveillance news story | 0.0895 | -0.111 | 0.066 |  | -3.082 | -3.729 | -0.035 |  | -4.110 | -4.993 | -0.030 |
|  | (4.303) | (4.308) | (0.054) |  | (3.036) | (3.028) | (0.038) |  | (3.074) | (3.070) | (0.039) |
|  |  |  |  |  |  |  |  |  |  |  |  |
| Moderator × surveillance news story | -5.775 | -5.329 | -0.131* |  | -3.455 | -2.235 | 0.000 |  | -0.574 | 1.861 | -0.020 |
|  | (4.429) | (4.434) | (0.055) |  | (4.037) | (4.026) | (0.050) |  | (3.908) | (3.903) | (0.049) |
|  |  |  |  |  |  |  |  |  |  |  |  |
| “New baby wellness” label | 3.752 | 8.460* | 0.030 |  | 7.881** | 8.823** | 0.004 |  | 5.390+ | 8.530** | -0.007 |
|  | (4.256) | (4.262) | (0.053) |  | (3.002) | (2.994) | (0.037) |  | (3.083) | (3.079) | (0.039) |
|  |  |  |  |  |  |  |  |  |  |  |  |
| Moderator × “new baby wellness” label | 6.379 | 4.145 | -0.041 |  | 2.416 | 8.316* | -0.008 |  | 8.383* | 7.633+ | 0.019 |
|  | (4.426) | (4.432) | (0.055) |  | (4.037) | (4.026) | (0.050) |  | (3.908) | (3.903) | (0.049) |
|  |  |  |  |  |  |  |  |  |  |  |  |
| *N* | 955 | 955 | 955 |  | 959 | 959 | 959 |  | 953 | 953 | 953 |
| Adjusted R-squared | 0.040 | 0.043 | 0.007 |  | 0.034 | 0.042 | 0.001 |  | 0.038 | 0.041 | -0.002 |

**Panel B: Moderation by help-seeking stigma, prior home visit experience, and discrimination in medical settings**

|  | Help-Seeking Stigma | | |  | Prior Home Visit Experience | | |  | Discrimination in Medical Settings | | |
| --- | --- | --- | --- | --- | --- | --- | --- | --- | --- | --- | --- |
|  | Interest in Learning More | Interest in Participating | Clicked on Link to Learn More |  | Interest in Learning More | Interest in Participating | Clicked on Link to Learn More |  | Interest in Learning More | Interest in Participating | Clicked on Link to Learn More |
| Moderator | -1.592 | -2.888 | -0.052 |  | 5.744 | 1.309 | -0.004 |  | -0.633 | 0.458 | 0.034 |
|  | (3.023) | (3.005) | (0.036) |  | (5.021) | (5.018) | (0.063) |  | (3.211) | (3.197) | (0.020) |
|  |  |  |  |  |  |  |  |  |  |  |  |
| Surveillance news story | -4.926+ | -4.787 | -0.017 |  | -4.659+ | -5.049+ | -0.038 |  | -6.172+ | -7.188* | -0.016 |
|  | (2.937) | (2.919) | (0.035) |  | (2.753) | (2.751) | (0.035) |  | (3.290) | (3.275) | (0.041) |
|  |  |  |  |  |  |  |  |  |  |  |  |
| Moderator × surveillance news story | 1.561 | 2.463 | -0.053 |  | 4.559 | 8.017 | 0.038 |  | 3.375 | 4.961 | -0.040 |
|  | (3.581) | (3.560) | (0.043) |  | (6.064) | (6.061) | (0.076) |  | (3.805) | (3.788) | (0.047) |
|  |  |  |  |  |  |  |  |  |  |  |  |
| “New baby wellness” label | 7.945** | 9.063** | 0.0075 |  | 8.232** | 10.91*** | 0.008 |  | 9.196** | 12.42*** | 0.036 |
|  | (2.951) | (2.934) | (0.035) |  | (2.759) | (2.758) | (0.035) |  | (3.216) | (3.202) | (0.040) |
|  |  |  |  |  |  |  |  |  |  |  |  |
| Moderator × “new baby wellness” label | 1.271 | 3.495 | 0.004 |  | 3.631 | 5.932 | -0.047 |  | -2.080 | -2.561 | -0.070 |
|  | (3.582) | (3.561) | (0.043) |  | (6.046) | (6.043) | (0.076) |  | (3.805) | (3.787) | (0.047) |
|  |  |  |  |  |  |  |  |  |  |  |  |
| *N* | 1093 | 1093 | 1093 |  | 960 | 960 | 960 |  | 971 | 971 | 971 |
| Adjusted R-squared | 0.035 | 0.034 | 0.012 |  | 0.044 | 0.046 | -0.002 |  | 0.033 | 0.038 | 0.001 |

*Note*: OLS regression models. The table shows coefficients with standard errors in parentheses.

+ *p* < .10, * *p* < .05, ** *p* <.01, *** *p* < .001

***Table I. Study 3 marginal effects of statistically significant pre-registered moderators***

| **Gender** | Effect of Surveillance News Story | SE | *P* |
| --- | --- | --- | --- |
| Female | -0.065 | (0.036) | .073 |
| Male | 0.066 | (0.054) | .219 |
|  |  |  |  |
| **Educational Attainment** | Effect of “New Baby Wellness” Label | SE | *P* |
| More than high school | 8.823 | (2.994) | .003 |
| High school degree or less | 17.139 | (3.775) | <.001 |
|  |  |  |  |
| **Financial Strain** | Effect of “New Baby Wellness” Label | SE | *P* |
| Never, rarely or only sometimes worry about meeting bills | 8.530 | (3.08) | .006 |
| Frequently worry about meeting bills | 16.162 | (3.590) | <.001 |

*Note*: Average marginal effects (dy/dx).

***Table J. Study 3 pre-registered regression analyses of mediation***

|  | (M1) | (M2) | (M3) | (M4) | (M5) | (M6) | (M7) |
| --- | --- | --- | --- | --- | --- | --- | --- |
|  | Trust-Related Obstacles to Engagement | Interest in Learning More | Interest in Learning More | Interest in Participating | Interest in Participating | Click-through | Click-through |
|  |  |  |  |  |  |  |  |
| Surveillance news story | 0.102 | -4.974+ | -3.936 | -5.000+ | -3.879 | -0.028 | -0.026 |
|  | (0.0701) | (2.658) | (2.615) | (2.657) | (2.601) | (0.034) | (0.034) |
|  |  |  |  |  |  |  |  |
| “New baby wellness” label | -0.217** | 8.975*** | 7.416*** | 11.94*** | 10.20*** | 0.007 | 0.002 |
|  | (0.0695) | (2.637) | (2.601) | (2.636) | (2.588) | (0.034) | (0.034) |
|  |  |  |  |  |  |  |  |
| Surveillance news story × “new baby wellness” label | 0.00620 | 5.746 | 5.543 | 1.182 | 0.986 | 0.046 | 0.046 |
|  | (0.0989) | (3.754) | (3.684) | (3.752) | (3.665) | (0.048) | (0.048) |
|  |  |  |  |  |  |  |  |
| Trust-related obstacles to engagement |  |  | -7.154*** |  | -7.991*** |  | -0.025 |
|  |  |  | (1.226) |  | (1.219) |  | (0.016) |
| *N* | 941 | 942 | 941 | 942 | 941 | 942 | 941 |
| Adjusted R-squared | 0.026 | 0.070 | 0.101 | 0.075 | 0.115 | 0.028 | 0.029 |

*Note*: OLS regression of treatment effects on the hypothesized mediator (M1), treatment effects on dependent variables without controlling for the mediator (M2, M4, M6), and treatment effects on dependent variables controlling for the mediator (M3, M5, M7). Interest in learning more and interest in participating in parenting programs are both scored from 0 (not at all interested) to 100 (very interested). Click-through is equal to 1 if the participant clicked the link to learn more, and 0 if not. All models include pre-registered covariates.

+ *p* < .10, * *p* < .05, ** *p* <.01, *** *p* < .001

***Table K. Study 3 pre-registered structural equation analysis of mediation***

|  | Coefficient (Standardized) | Robust SE | *z* | *P* > \|*z*\| | 95% Confidence Interval | |
| --- | --- | --- | --- | --- | --- | --- |
| ***Structural Model Pathways*** |  |  |  |  |  |  |
| Surveillance news story 🡪 Trust-related obstacles to engagement | 0.044 | 0.030 | 1.450 | 0.148 | -0.015 | 0.103 |
| “New baby wellness” label 🡪 Trust-related obstacles to engagement | -0.120 | 0.030 | -3.970 | 0.000 | -0.178 | -0.061 |
|  |  |  |  |  |  |  |
| Trust-related obstacles to engagement 🡪 Interest in learning more | -0.168 | 0.030 | -5.550 | 0.000 | -0.228 | -0.109 |
| Surveillance news story 🡪 Interest in learning more | -0.018 | 0.029 | -0.620 | 0.533 | -0.076 | 0.039 |
| “New baby wellness” label 🡪 Interest in learning more | 0.167 | 0.029 | 5.780 | 0.000 | 0.110 | 0.224 |
|  |  |  |  |  |  |  |
| Trust-related obstacles to engagement 🡪 Interest in participating | -0.183 | 0.031 | -5.990 | 0.000 | -0.243 | -0.123 |
| Surveillance news story 🡪 Interest in participating | -0.045 | 0.029 | -1.550 | 0.122 | -0.102 | 0.012 |
| “New baby wellness” label 🡪 Interest in participating | 0.164 | 0.029 | 5.750 | 0.000 | 0.108 | 0.220 |
|  |  |  |  |  |  |  |

| ***Direct, Indirect, and Total Effects*** |  |  |  |  |  |  |
| --- | --- | --- | --- | --- | --- | --- |
| Direct effects |  |  |  |  |  |  |
| *Independent variables to mediator* | Coefficient | Robust SE | *z* | *P* > \|*z*\| | 95% Confidence Interval | |
| Surveillance news story 🡪 Trust-related obstacles to engagement | 0.066 | 0.045 | 1.450 | 0.148 | -0.023 | 0.155 |
| “New baby wellness” label 🡪 Trust-related obstacles to engagement | -0.180 | 0.045 | -3.960 | 0.000 | -0.269 | -0.091 |
| *Independent variables and mediator to dependent*  *variable 1* |  |  |  |  |  |  |
| Trust-related obstacles to engagement 🡪 Interest in learning more | -6.615 | 1.194 | -5.540 | 0.000 | -8.956 | -4.275 |
| Surveillance news story 🡪 Interest in learning more | -1.083 | 1.738 | -0.620 | 0.533 | -4.490 | 2.323 |
| “New baby wellness” label 🡪 Interest in learning more | 9.885 | 1.733 | 5.700 | 0.000 | 6.487 | 13.282 |
| *Independent variables and mediator to dependent*  *variable 2* |  |  |  |  |  |  |
| Trust-related obstacles to engagement 🡪 Interest in participating | -7.153 | 1.200 | -5.960 | 0.000 | -9.506 | -4.801 |
| Surveillance news story 🡪 Interest in participating | -2.650 | 1.716 | -1.540 | 0.122 | -6.013 | 0.713 |
| “New baby wellness” label 🡪 Interest in participating | 9.655 | 1.710 | 5.650 | 0.000 | 6.303 | 13.007 |
|  |  |  |  |  |  |  |
| Indirect effects |  |  |  |  |  |  |
| *On dependent variable 1* | Coefficient | Robust SE | *z* | *P* > \|*z\|* | 95% Confidence Interval | |
| Surveillance news story via trust-related obstacles to engagement | -0.435 | 0.314 | -1.390 | 0.166 | -1.050 | 0.180 |
| “New baby wellness” label via trust-related obstacles to engagement | 1.189 | 0.383 | 3.110 | 0.002 | 0.440 | 1.939 |
| *On dependent variable 2* |  |  |  |  |  |  |
| Surveillance news story via trust-related obstacles to engagement | -0.470 | 0.339 | -1.390 | 0.165 | -1.134 | 0.194 |
| “New baby wellness” label via trust-related obstacles to engagement | 1.286 | 0.406 | 3.170 | 0.002 | 0.490 | 2.082 |
|  |  |  |  |  |  |  |
| Total effects |  |  |  |  |  |  |
| *On dependent variable 1* | Coefficient | Robust SE | *z* | *P* > \|*z\|* | 95% Confidence Interval | |
| Surveillance news story 🡪 Interest in learning more | -1.518 | 1.761 | -0.860 | 0.389 | -4.970 | 1.933 |
| “New baby wellness” label 🡪 Interest in learning more | 11.074 | 1.760 | 6.290 | 0.000 | 7.624 | 14.524 |
| *On dependent variable 2* |  |  |  |  |  |  |
| Surveillance news story 🡪 Interest in participating | -3.120 | 1.743 | -1.790 | 0.073 | -6.536 | 0.296 |
| “New baby wellness” label 🡪 Interest in participating | 10.941 | 1.741 | 6.280 | 0.000 | 7.528 | 14.354 |

*Note*: Structural equation model estimated using maximum likelihood with robust standard errors. Model includes covariance between learning more and participating (not shown). Model fit: $\chi_{2}$(9) = 1,559.20, *p* < 0.001; AIC = 2,5161.53; BIC = 2,5236.50; CFI = 1.00; TLI = 1.00; RMSEA = 0.000; SRMR = 0.000.

# **Supporting Information References**

1. J. E. Dumas, J. Nissley-Tsiopinis, A. D. Moreland, From intent to enrollment, attendance, and participation in preventive parenting groups. *J. Child Fam. Stud.* **16**, 1–26 (2007).

2. D. L. Vogel, N. G. Wade, S. Haake, Measuring the self-stigma associated with seeking psychological help. *J. Couns. Psychol.* **53**, 325–337 (2006).

3. R. E. Brenner, K. F. Colvin, J. H. Hammer, D. L. Vogel, Using item response theory to develop revised (SSOSH-7) and ultra-brief (SSOSH-3) Self-Stigma of Seeking Help scales. *Assessment* **28**, 1488–1499 (2021).

4. Z. Hill, M. Spiegel, L. A. Gennetian, Pride-based self-affirmations and parenting programs. *Front. Psychol.* **11**, 1–9 (2020).

5. N. Krieger, K. Smith, D. Naishadham, C. Hartman, E. M. Barbeau, Experiences of discrimination: Validity and reliability of a self-report measure for population health research on racism and health. *Soc. Sci. Med.* **61**, 1576–1596 (2005).

6. D. R. Williams, Y. Yu, J. S. Jackson, N. B. Anderson, Racial differences in physical and mental health: Socio-economic status, stress and discrimination. *J. Health Psychol.* **2**, 335–351 (1997).
